# Characterization of the complete chloroplast genome of *Poa poophagorum* (Gramineae), a native grass from the Qinghai-Tibetan Plateau

**DOI:** 10.1080/23802359.2020.1719925

**Published:** 2020-01-31

**Authors:** Linna Wei, Chunping Zhang, Quanmin Dong, Yang Yu, Xiaoxia Yang

**Affiliations:** Qinghai Academy of Animal and Veterinary Science, State Key Laboratory of Plateau Ecology and Agriculture in the Three River Head Waters Region, Qinghai University, Xining, Qinghai, China

**Keywords:** *Poa poophagorum*, native grass, complete chloroplast genome, Illumina sequencing, phylogenetic analysis

## Abstract

This study provides a chloroplast genome of *Poa poophagorum*. The complete cp genome was135,664 bp in length with typical quadripartite structure, containing a pair of inverted repeats (IR) of 21,552 bp each, a large single-copy (LSC) region of 79,790 bp, and a small single-copy (SSC) region of 12,770 bp. The overall G + C content of the cp genome was 38.30%, which encompassed 119 genes including 79 protein-coding genes,8 rRNA genes, and 32 tRNA genes. The phylogenetic analysis indicated that *P. poophagorum* was closely related to *Festuca arundinacea* cultivar KY-31in Gramineae. This study would contribute to enrich the *Poa* L. cp genome resource and promote biological research.

*Poa* L. is one the largest genus in Gramineae, it is known that there are 400 species of *Poa* L. in the world, mostly in temperate and cold regions, and rarely in the tropics (Yang-Chun 2002). There are about 100 species of *Poa* L. in China, which are mainly distributed in the northern regions, among these, 36 species are found in Qinghai-Tebitan Plateau (Sun 2002). *Poa poophagorum* is perennial or annual grass of Gramineae, distributed in the altitude of 3000 ∼ 5500 m in Qinghai-Tebitan Plateau(Zhang et al. 2014). It has a strong regeneration capacity and tillering ability, it also exhibits better palatability to animals (Guan et al. 2011). Therefore, in this study, we conducted a high-throughput sequencing analysis of the complete chloroplast (cp) genome of *P. poophagorum*, and provide valuable information to study the evolution of the genus *Poa* L.

In this study, the seeds of *P. poophagorum* were collected from Maqin county, Qinghai Province, China (33°43′∼35°16′N, 98°48′∼100°55′E). *Poa poophagorum* seeds are stored in the Key Laboratory of Superior Forage Germplasm in the Qinghai-Tibetan Plateau, the specimen Accession number is ‘BF1.’ Total genomic DNA was extracted from fresh leaves using a CTAB DNA-extraction protocol. The cp genome was assembled using NovoPlasty (version: 3.6; k-mer = 39; Dierckxsens et al. 2017), which was annotated using the software CpGAVAS (Liu et al. 2012). The phylogenetic tree analysis was determined using MEGA X (Kumar et al. 2018) with the neighbor-joining algorithm. The sequence of whole cp genomes was annotated utilizing the software Geneious R8.0.2 (Biomatters Ltd., Auckland, New Zealand), and was deposited in GenBank (accession numbers MN551181).

The complete cp genome sequence of *P. poophagorum* was 135,664 bp in length, which consisted of 78 protein-coding genes, 8 rRNA genes, and 32 tRNA genes. The sequence also contained a pair of inverted repeat regions (IR; 21,552 bp each), a large single-copy region (LSC; 79,790 bp), and a small single-copy region (SSC; 12,770 bp, Figure 1). The average GC content of the overall genome was 38.30%, compared with 36.25% for LSC, 43.82% for IRA, 32.48% for SSC, and 43.82% for IRB, respectively. Among these genes, there are six protein-coding genes (*rpl23*, *rps7*, *rps15*, *rps3*, *rps22*, and *rps19*), six transfer RNA genes (*trnI-CAU*, *trnN-GUU*, *trnR-ACG*, *trnI-GAU*, *trnV-GAC*, and *trnL-CAA*), four ribosomal RNA genes (*rrn16*, *rrn4.5*, *rrn23*, and *rrn5*) duplicated in IR regions.

**Figure 1.**
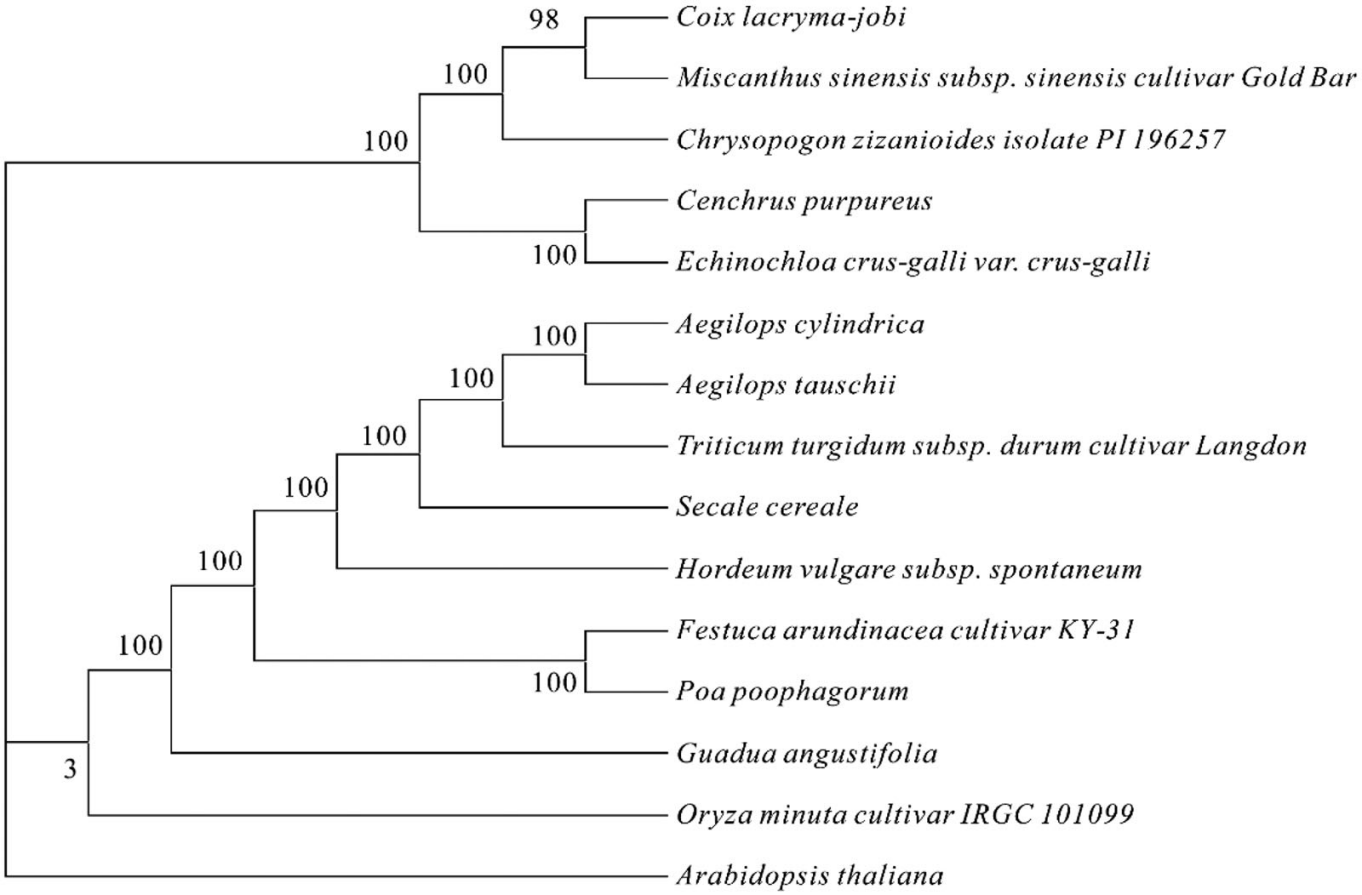
Maximum-likelihood phylogenetic tree based on 15 selected Gramineae complete chloroplast genome sequences.

To further validate the assembled sequence, we presented the phylogenetic estimation with MrBayes (Ronquist and Huelsenbeck 2003) for *P. poophagorum* based on molecular data of relatively fifteen related cp genomes from GenBank database, using all CDS sequences, GTR model, 100,000 generation, and gamma rates across sites, and yielded a well-supported phylogenetic estimation for Pedicularis with 100% bootstrap support (Figure 1). The phylogenetic analysis showed that *P. poophagorum* was more closely related to the reported species of *Festuca arundinacea cultivar KY-31* in Gramineae. The complete cp genome of *P. poophagorum* would provide more valuable information to study the evolution of genus *Poa* L.

## References

[CIT0001] Dierckxsens N, Mardulyn P, Smits G. 2017. NOVOPlasty: de novo assembly of organelle genomes from whole genome data. Nucleic Acids Research. 45(4):e18. 28204566 10.1093/nar/gkw955 PMC5389512

[CIT0002] Guan FC, Miao YJ, Wang C. Luosangwangjiu C. 2011. Turf characteristics of wild *Poa poiphagorum* and its application in Tibet. Pratacult Sci.7(1):1259–1262.

[CIT0003] Kumar S, Stecher G, Li M, Knyaz C, Tamura K. 2018. MEGA X: Molecular Evolutionary Genetics Analysis across computing platforms. Mol Biol Evol. 35(6):1547–1549. 29722887 10.1093/molbev/msy096 PMC5967553

[CIT0004] Liu C, Shi L, Zhu Y, Chen H, Zhang J, Lin X, Guan X. 2012. CpGAVAS, an integrated web server for the annotation, visualization, analysis, and GenBank submission of completely sequenced chloroplast genome sequences. BMC Genomics. 13(1):715. 23256920 10.1186/1471-2164-13-715 PMC3543216

[CIT0005] Ronquist F, Huelsenbeck JP. 2003. MrBayes 3: Bayesian phylogenetic inference under mixed models. Bioinformatics. 19(12):1572–1574. 12912839 10.1093/bioinformatics/btg180

[CIT0006] Sun HQ. 2002. Biodiversity analysis of Gramineae plants in Qinghai province, China. Pratacult Sci. 19:7–12.

[CIT0007] Yang-Chun LI. 2002. RAPD analysis on inter-species relationships in *Poa*. Acta Pratacult Sci. 11:94–99.

[CIT0008] Zhang Z, Niu K, Liu X, Jia P, Du G. 2014. Linking flowering and reproductive allocation in response to nitrogen addition in an alpine meadow. J Plant Ecol. 7(3):231–239.

